# Psychedelic therapies reconsidered: compounds, clinical indications, and cautious optimism

**DOI:** 10.1038/s41386-023-01656-7

**Published:** 2023-07-21

**Authors:** Jennifer M. Mitchell, Brian T. Anderson

**Affiliations:** 1 Department of Neurology, University of California San Francisco, San Francisco, CA USA; 2 Department of Psychiatry and Behavioral Sciences, University of California San Francisco, San Francisco, CA USA; 3 Department of Veterans Affairs, Research Service, San Francisco VA Medical Center, San Francisco, CA USA; 4 Berkeley Center for the Science of Psychedelics, University of California Berkeley, Berkeley, CA USA

**Keywords:** Neuroscience, Psychology

## Abstract

The clinical investigation of psychedelic medicines has blossomed over the last 5 years. Data from a Phase 3 industry trial and a multicenter Phase 2 industry trial, in addition to multiple early phase investigator-initiated and industry trials, have now been published in peer-reviewed journals. This narrative review summarizes both the recent data and the current clinical trials that are being conducted with various classes of “psyche-manifesting” substances, which may prove beneficial in the treatment of a broad range of conditions. Methodological considerations, unique challenges, and next steps for research are discussed in keeping with the uniquely “experiential” nature of these therapies.

## Introduction

The past few years have ushered in a renewed wave of psychedelic interest and research that has led to the clinical testing of multiple psychedelic agents for various health conditions. Australia and Canada have even recently approved the clinical use of certain psychedelic medicines under restricted circumstances [1, 2]. However, along with this renewed wave of interest has come a series of challenges with respect to the taxonomy of psychedelics, the implementation of best practices for the evaluation of psychedelic therapies, and the capacity to stay abreast of developments in this rapidly expanding scientific field. Here we review the contemporary clinical research to provide an overview of the different classes of psychedelics currently under investigation for clinical use, their proposed indications, and—because they are “experiential medicines”—considerations for their judicious use.

Psychedelics have long been implicated in the treatment of mood and anxiety disorders, as well as disorders related to impulsivity, repetitive behaviors, and impaired decision-making, such as alcohol and other substance use disorders [3–5]. Yet the clinical promise of mid-20th Century psychedelic research was ultimately overshadowed by methodological limitations [6], regulatory restrictions, and a political landscape that made psychedelic research all but untenable for several decades [7]. Current biomedical theories speculate that psychedelic therapies improve clinical functioning by regulating affective states (such as anhedonia) and self-referential cognitive processes and may therefore ultimately involve interrelated neural circuits across a broad range of conditions [8, 9]. Along these lines, the current clinical research landscape has expanded significantly compared to even a few years ago [10], and now includes evaluations of the effects of psychedelic therapies in a wide array of diagnostic categories, including mood disorders, substance use disorders, obsessive compulsive and related disorders, trauma-related disorders, and disorders of dysfunctional coping such as pathological grief and end of life distress. Typically, when investigated as therapies for psychiatric conditions, psychedelics are administered as adjuncts to a brief course of behavioral therapy to mitigate the risk of adverse events and to augment efficacy [11, 12].

In addition to psychiatric indications, psychedelics are also being studied more generally as neuroplastic agents, potentially capable of inducing change in intractably crystalized neurological pathways. As such, they are being pursued as therapeutics for age-related degenerative conditions—including Alzheimer’s and Parkinson’s—as well as for pain, headache and migraine, autism, and visual impairments. Only time will tell if these compounds can reverse ingrained neurological processes, perhaps by enabling neuroplasticity, to the point where previously inflexible systems can be adjusted and reset.

### Defining psychedelic

The original definition of the term *psychedelic*, as coined by the psychiatrist Humphry Osmond, is “mind-” or “soul-manifesting”, and as such is independent of biological mechanism. With this definition in mind, drugs that activate the serotonin 5-HT2A receptor directly (typically termed classic psychedelics) [13] as well as those that activate serotonin receptors indirectly or not at all, or that work through binding to a combination of receptors (including glutamatergic, dopaminergic, and opioidergic receptors) would all be considered psychedelic if they demonstrate the capacity for allowing greater access to the *psyche* in a manner similar to the classic psychedelics. Therefore, this review will include a series of drugs that meet the original definition of *psychedelic* for which there exists preliminary evidence from modern trials of the potential to affect psychotherapeutic change in humans. Some *atypical, or non-classic, psychedelics* are also included here; these are drugs with psychoactive effects that are not primarily or uniquely mediated through the activation of the 5-HT2A receptor. The decades-long history of the use of substances like ketamine [14, 15] and psychedelic amphetamines like 3,4-methelenedioxyamphetamine (MDA) [16] in psychedelic therapy suggests that a comprehensive review of psychedelic therapies should include drugs beyond the classic psychedelics.

While the term *hallucinogen* is often colloquially substituted for the term psychedelic, hallucinogens are a category of drugs that induce perceptions in the absence of external stimuli (as opposed to inducing perceptual distortions of extant stimuli). Therefore, although there is some overlap in the categories of hallucinogen and psychedelic, here we will only use the term psychedelic, as we are summarizing effects that are more broadly relevant to processes of psychotherapeutic change, and not limited to perceptual alterations per se. With these definitions in mind, and considering the available data and currently registered trials, the modern clinical investigation of the following classic and atypical psychedelics (henceforth, collectively referred to as “psychedelics”) will be discussed in this review: ayahuasca, N,N-dimethyltrypatmine (DMT) and 5-methoxy-DMT (5-MeO-DMT), ibogaine, ketamine, lysergic acid diethylamide (LSD), 3,4-methylenedioxymethamphetamine (MDMA), mescaline, salvinorin A, and psilocybin. In addition, as psychedelics are thought to be “experiential medicines” affected by both set and setting [17–19], attention will also be focused on the environmental and psychological conditions that modulate the therapeutic efficacy of psychedelics.

Many psychedelic molecules have structural similarities that may enable specific signaling mechanisms. However, molecular differences allow them to be divided into indolamines (including the ergolines and simple tryptamines), phenethylamines, diterpines, and cyclohexanones. Each of these will be discussed in turn in an attempt to provide the reader an up-to-date view of the rapidly evolving field of clinical research into *psyche*-manifesting drugs.

## Indolamines

### Ibogaine

Ibogaine is an indolamine that is derived from the West African *Tabernanthe iboga* bush that has long been used as part of the Bwiti religious tradition in the jungles of Gabon [20]. Ibogaine binds dopamine and serotonin (5-HT) receptors, acts as both an NMDA and a3b4-nicotinic receptor antagonist, and acts as a kappa opioid receptor agonist [21]. Although ibogaine was originally touted as an anti-addiction medication as far back as the 1960s https://www.nytimes.com/2010/02/17/us/17lotsof.html, it took over 30 years for it to make its way into phase 1 clinical studies, which indicated that, although ibogaine has the potential to be an effective an anti-addiction therapeutic for a number of different substance use disorders [22–24], it also carries cardiac and neurological risks that complicate its use as a therapeutic [25–27]. A series of sudden and unexpected deaths halted phase 1 testing for drug abuse and dependence [25, 28] and clinical trials have yet to resume in the United States, although there is growing political support for these studies [29].

Although ibogaine is a Schedule 1 substance and is not FDA approved, over the last few decades it has been administered at drug and alcohol treatment centers in Latin America and the Caribbean, and it is currently under investigation outside of the United States as a potential therapeutic for alcohol misuse (NCT03380728), and for drug use, dependence, and withdrawal (NCT04003948; NCT05029401). Recent data suggest that the potential negative impact of ibogaine on cardiac function can be controlled through careful screening and monitoring during drug administration [30] and that, as both ibogaine and methadone may induce QT prolongation (an alteration in cardiac ventricular repolarization that is associated with the Torsade de Pointes arrhythmia and increased risk of sudden cardiac death), care should be taken to ensure that those seeking ibogaine treatment for opioid use disorder are screened for the use of methadone and other QT prolonging drugs prior to ibogaine administration.

Elucidation of the mechanism(s) by which ibogaine exerts its clinical effects might lend insight into the contribution of various neurotransmitter systems to the clinical effectiveness of psychedelics, as ibogaine is perhaps one of the least pharmacologically specific, and yet most impactful, psychedelics currently under investigation.

## Indolamines: Ergolines

### Lysergic acid diethylamide (LSD)

LSD was first synthesized by the chemist Albert Hoffman during his employment with Sandoz Pharmaceuticals in the 1930s while he was searching for novel compounds to treat respiratory depression. LSD has since been shown to bind with high affinity to several 5-HT receptors [31] and also acts as a dopamine (D1, D2, D4) receptor agonist [32, 33]. Potential clinical indications include alcohol and other substance use disorders [34–36], obsessive compulsive disorder [37], depression (now in phase 2), end of life anxiety [38], cluster headache [39], and attention-deficit hyperactivity disorder (ADHD) [40]. LSD has long been available through Compassionate Use laws in Switzerland as an adjunct to psychotherapy for the treatment of a number of different mental health conditions [41].

Although a substantial volume of data from the 1960s to 1970s suggest that LSD could be effective in decreasing alcohol consumption in self-ascribed, and self-selected, “alcoholics,” [42] and although a more recent meta-analysis that incorporated clinical data from 6 smaller studies demonstrated that a single dose of LSD resulted in a long-term decrease in alcohol “misuse,” [35] previous outcome measures were not collected using current methodological standards and should therefore be replicated to confirm and extend previous findings in a thoroughly characterized subject population with alcohol use disorder (AUD). Fortunately, a double-blind, placebo-controlled, randomized, multisite study is currently being planned (NCT05474989) to evaluate the effects of two doses of LSD (150 µg for first dose followed by either 150 µg or 250 µg for second dose) on prevention of relapse to alcohol in a population with AUD. These data will hopefully provide insight into whether LSD does indeed hold promise as a potential therapeutic for AUD.

LSD has also recently completed early phase clinical testing for anxiety disorders (NCT03153579; NCT00920387) and the results have shown that LSD decreases anxiety while increasing quality of life [43] and also, importantly, that these effects are long-lasting [38]. In addition, a randomized, double-blind, placebo-controlled phase 2 trial of LSD for major depression (comparing two moderate to high doses of LSD = 100 µg/100 µg or 100 µg/200 µg, and two low doses of LSD = 25 µg/25 µg) has now been completed (NCT03866252), although these data were not yet available for review at the time of this publication. Lastly, a multisite, randomized, double-blind, placebo-controlled phase 2 trial is also currently being planned to further study the use of LSD for adults with ADHD (NCT05200936).

In addition to the mental health disorders listed above, both case (40. Sewell et al., 2006) and self-reports [44] indicate that LSD could be effective in combating cluster headache, and a double-blind, randomized, placebo-controlled phase 2 trial of LSD (3 doses of 100 mcg over 3 weeks) has recently been initiated to evaluate the efficacy of LSD for treatment of cluster headache (NCT03781128). Finally, as part of a drug development program for treating Alzheimer’s Disease, a recent double-blind randomized pilot trial of repeated (every 4 days) low doses of LSD (5 mcg vs 10 mcg vs 20 mcg vs placebo) in healthy older adults (age 55–75) found the drug to be well-tolerated in this population [45].

## Indolamines: Simple tryptamines

### Psilocybin

Psilocybin is an active agent in *Psilocybe* mushrooms, which have been used ritualistically for thousands of years by Indigenous communities in Central and North America. Psilocybin exerts its psychedelic effects primarily through activation of 5HT2A receptors [46] and through activation of a 5-HT2AR-mGluR2 receptor complex [47]. Likely due to its well-established safety profile, minimal abuse potential, and short duration of subjective perceptual effects, psilocybin is currently the most broadly studied psychedelic for mental health conditions. Clinical indications under investigation currently include major depression [48], treatment-resistant depression [49], alcohol and other substance use disorders [50], smoking cessation [51], and OCD [52]. Psilocybin has also been studied in conjunction with individual and group psychotherapy for treating distress in patients with serious illness including cancer-related mood and anxiety disorders, and demoralization in long term AIDS survivors [53].

Not only has psilocybin been successfully administered for smoking cessation [51, 54], but, intriguingly, it has also been shown that change in tobacco consumption following psilocybin administration is correlated with the degree of “mystical-type experience” reported by study participants, such that those reporting greater intensity of mystical-type experiences also report a greater decrease in smoking [54]. While there remains great debate over the nature and assessment of a “mystical-type experience”, high-dose psilocybin has repeatedly been reported by participants to be a spiritual or transcendent event, which seems to be an important contributor to treatment effectiveness [55] and, as such, merits further attention.

A modest-sized proof of principle phase 2 trial recently demonstrated the significant and long-lasting efficacy of psilocybin when combined with psychotherapy for the treatment of AUD (**50**. Bogenschutz et al., 2022). Mechanistic (NCT04141501), head-to-head (NCT05421065), larger, multisite (NCT05646303), and other trials (NCT04620759) of psilocybin therapy for AUD are currently recruiting.

The most detailed exploration of psilocybin for a therapeutic indication thus far has been for treatment-resistant depression (TRD), and a recent, double-blind phase 2 study found a dose-dependent reduction in depression scores in the weeks following administration of a single dose of psilocybin (1 mg, 10 mg, or 25 mg) [56]. Similarly, a trial investigating the use of two psilocybin administration sessions in conjunction with therapy for major depressive disorder (MDD) not only found a significant attenuation in depression scores at both the primary endpoint and at the 4-week follow up [57] but also noted that these effects were still durable 1 year after psilocybin administration [58]. This trial also noted a correlation between mystical-type experience at the time of psilocybin administration and increased well-being at the 12-month follow-up. As multiple studies have noted a positive correlation between the lasting impact of psilocybin on mental health measures and mystical-type experiences, it will be interesting to note whether future studies will be able to elucidate the nature of the relationship between psilocybin-induced mystical-type experiences and durable alleviation of mental health conditions.

A myriad of additional phase 2 trials with psilocybin are now underway for a variety of other indications including PTSD (NCT05554094; NCT05243329; NCT05312151), OCD (NCT05370911; NCT04882839; NCT03300947; NCT05546658; NCT03356483), depression in bipolar 2 disorder (NCT0506529; NCT04433845), anorexia nervosa (NCT04656301; NCT04052568; NCT04661514; NCT05481736; NCT04505189), binge eating (NCT05035927), fibromyalgia (NCT05548075; NCT05128162; NCT05068791), phantom limb pain (NCT05224336), migraine (NCT03341689; NCT04218539), cluster headache (NCT02981173), concussion headache (NCT03806985). Multiple trials are assessing psilocybin therapy for distress associated with serious medical illness (NCT04950608; NCT05398484; NCT05506982; NCT04522804; NCT05220046; NCT04593563; NCT05403086). In addition, a series of studies have been evaluating the potential of psilocybin to attenuate methamphetamine use disorder (NCT04982796; NCT05322954) and cocaine use disorder (NCT02037126). With any luck, the next couple of years should further quantify the myriad of potential therapeutic uses of psilocybin.

### Ayahuasca

Ayahuasca is typically an admixture of the *Banisteriopsis caapi* vine, containing MAO-inhibiting beta-carboline alkaloids, and the DMT-containing leaves of the *Psychotria viridis* shrub, although other plants, such as *Diplopterys cabrerana*, are at times used to make decoctions that are also referred to as ayahuasca. The drink has been used ceremonially in the Amazon Basin for at least hundreds of years and is used widely today in shamanic and other religious contexts within and outside of South America [59]. Potential indications for ayahuasca include alcohol and other substance use disorders, anxiety and depression disorders [60] and possibly prolonged grief disorder [61] and eating disorders [62]. Naturalistic studies have indicated that regular users of ayahuasca consume less alcohol and other drugs compared to other populations [63–65], and that ritual participants self-report improved affective symptoms after drinking ayahuasca [66], however individuals with anxiety and mood disorders may also be at higher risk of experiencing adverse effects in rituals settings [67]. One recent placebo-controlled proof of principle trial has also shown that a single administration of ayahuasca can attenuate symptoms of treatment-resistant depression [68].

### DMT & 5-MeO-DMT

N,N-Dimethyltryptamine (DMT) is a substituted tryptamine that constitutes one of the primary active ingredients in ayahuasca and is structurally similar to the psychedelic compounds 5-MeO-DMT and bufotenin (5-HO-DMT). In addition to high binding affinity at a number of 5-HT receptors, DMT acts as a TAAR agonist [69], and a sigma receptor agonist [70] and may mediate effects at metabotropic glutamate receptors [71]. Although the clinical data are currently limited, DMT is now being studied in a fixed order, open-label, dose-escalation study in participants with major depression (NCT04711915), and a double-blind, randomized, placebo-controlled study of intravenous DMT in subjects with major depressive disorder (MDD) has now been completed (NCT04673383). Because the subjective effects of DMT are short-lasting compared to other psychedelic compounds [72], DMT might lend itself more readily to use in clinical settings.

## Phenethylamines

### MDMA

3,4-Methylenedioxymethamphetamine (MDMA) was originally synthesized by the pharmaceutical company Merck in 1912 as part of a research program on anticoagulating agents. Early case reports suggested that MDMA could be a remarkably effective catalyst in both individual and couples psychotherapy [73, 74] for a variety of psychological issues. The psychedelic-like effects of MDMA were eventually immortalized by Alexander and Ann Shulgin in their book, PiHKAL [75].

MDMA acts on human monoamine transporters [76], though most of the subjective effects of MDMA are dependent on serotonin release, which MDMA potentiates through a series of different mechanisms. MDMA inhibits the 5-HT vesicular transporter (VMAT2) and activates the intracellular presynaptic terminal receptor (TAAR1), which impacts both the release and reuptake of serotonin [69, 77, 78]. Downstream of serotonin efflux, MDMA promotes the release of oxytocin [79], a neuromodulator shown to play a critical role in bonding and social interactions [80], which may therefore facilitate the therapeutic process by enabling participants to remain emotionally open while they explore difficult memories and subject matter.

MDMA most likely exerts its influence through effects within the amygdala, and previous human research indicates that MDMA attenuates left amygdalar responses to angry facial expressions and enhances ventral striatal responses to happy expressions [81]. More recent research has found that, when administered to subjects with severe PTSD, MDMA induces changes in functional connectivity between the left amygdala and both the left insula and bilateral posterior cingulate cortex during autobiographical memory recall [82]. Further experiments are needed to address individual differences in responsivity to MDMA and to determine if and how to maximize the effects of MDMA administration on retrieval and reconsolidation of negative memories.

MDMA is typically administered in conjunction with therapy and the combination of MDMA plus therapy is has recently been investigated for use in indications including PTSD [83, 84], social anxiety in adults with [85] and without autism (NCT05138068), AUDs (NCT05709353), illness-related anxiety (NCT02427568), adjustment disorder (NCT05584826), fear extinction (NCT03527316), and eating disorders (NCT04454684). The most thoroughly investigated of these indications is currently PTSD. Indeed, MDMA for PTSD might well be the first psychedelic to be submitted to the FDA as part of a new drug application (NDA) for regulatory approval and is the only psychedelic to date to have completed phase 3 clinical trials. Phase 3 findings demonstrated that MDMA-therapy was both safe and effective in treating PTSD, functional disability, and symptoms of depression in a population with severe PTSD [83].

In addition to the current manualized inner-directed therapy that has been used in conjunction with MDMA administration in phase 3, several other studies are now underway to investigate the pairing of MDMA with other gold standard, manualized therapies for PTSD. Studies are being conducted to investigate the combination of MDMA plus exposure therapy for PTSD (NCT05746572), MDMA plus group therapy for Veterans with PTSD (NCT05173831), MDMA plus cognitive processing therapy for PTSD (NCT05067244), and MDMA plus cognitive behavioral conjoint therapy for couples with PTSD (NCT02876172). Also, because of its ability to potentiate self-compassion [86], MDMA could be particularly powerful in those suffering from moral injury in relation to PTSD.

### Mescaline

Mescaline is currently found in four species of cacti: Bolivian Flame, Peruvian Flame, San Pedro, and Peyote, the last of which has been used in ritual by Native American communities for thousands of years [87]. It has long been used as a treatment for alcoholism within Native American communities [88, 89].

Recent self-reported data (via an online questionnaire) indicate that mescaline may attenuate symptoms of anxiety, PTSD, depression, and both alcohol and substance use [90, 91]. In keeping with studies into the mechanistic actions of psilocybin, many participants rated their experience with mescaline as one of the most spiritually significant and meaningful experiences of their lives [91]. In addition, improvements in symptoms of anxiety, PTSD, depression, and both alcohol and other substance use were associated with greater “intensity of insight”, again demonstrating that some aspect of the subjective effect of the psychedelic experience is linked to clinical outcome. While clinical research with mescaline is still in its infancy, the data thus far suggest that mescaline may hold similar promise to other phenethylamines for the treatment of multiple mental health disorders.

## Diterpines

### Salvinorin A

*Salvia divinorum* is a sage species that is used ritualistically among the Mazatec tribe of Mexico. The active constituent, salvinorin A, is a kappa opioid agonist that has no discernable action at the 5HT_2A_ receptor, giving pause to the assertion that all psychedelics act primarily through 5HT_2A_ receptor activation. As a kappa agonist, salvinorin A may also hold clinical potential as a treatment for pain, ischemia, cardiac damage, and addiction [92], perhaps especially in biological females who do not find kappa agonists particularly aversive [93]. While there is still a paucity of human research on salvinorin A, recent findings indicate that when smoked, salvinorin A produces intense but short acting hallucinations and out of body experiences but, notably, no significant changes in heart rate or blood pressure [94]. In keeping with the classic psychedelics, administration of salvinorin A has also been shown to reduced brain wide dynamic functional connectivity (most notably in the default mode network), while increasing between-network static functional connectivity [95]. Unlike ibogaine, which also activates kappa opioid receptors and demonstrates anti-addictive properties, salvinorin A has not, to date, been shown to induce the notable adverse effects that have curtailed ibogaine’s development as a clinical therapeutic, and emergent events are rare (https://calpoison.org/news/salvia-divinorum). Because of its potential safety and novel pharmacological mechanism of action, further effort should be made to evaluate the pharmacological potential of salvinorin A.

## Dissociative agents (Cyclohexanones)

### Ketamine

Ketamine is a selective NMDA antagonist that has long been used as an anesthetic and animal tranquilizer, and which has recently found new use as a fast-acting—albeit temporary—treatment for depression [96]. While ketamine is best considered an atypical psychedelic, or perhaps a drug with psychedelic-like effects, the mechanism of action of ketamine (NMDA antagonism) is a contributor to the effects of several classic psychedelics (such as ibogaine and DMT) and may prove relevant to the further development of psychedelics as therapeutics. It is therefore worth briefly mentioning the state of current research with ketamine.

In addition to its use as an antidepressant, ketamine might hold promise for the treatment of anxiety and PTSD. A recent review indicates that, under certain circumstances (e.g., specified dose and route of administration), ketamine is effective in temporarily attenuating some anxiety disorders [97] and may therefore merit further investigation. With respect to PTSD symptomology, although a randomized, double-blind active-placebo-controlled trial demonstrated that 2 weeks of 3× weekly ketamine infusions are efficacious for up to a month in those with severe PTSD [98], a larger trial found no significant effect of 4 weeks of 2× weekly ketamine infusions in a population of military Veterans and service members with comorbid depression and PTSD [99]. Furthermore, recent years have seen the publishing of promising data on the use of ketamine as a rapid-acting therapy for substance use disorders and other neuropsychiatric conditions like obsessive-compulsive disorder [100]. As with previous depression trials, even if initially efficacious under certain dosing regimens, the limited durability of positive clinical outcomes of ketamine complicates drug administration and adoption as a frontline therapeutic [101] for a number of conditions. It is possible that, as with MDMA and psilocybin, durability of ketamine’s effects on mental health indications could be potentiated with the addition of psychotherapy and, to this end, a recent study has found that an automated, computerized training protocol might extend the effects of ketamine on depression [102]. Additional data are needed to determine whether and how the coupling of ketamine administration with psychotherapy [103] renders the drug more efficacious and durable for different indications.

## Set and setting are key variables

Set and setting have long been recognized as fundamental elements driving the clinical outcomes of psychedelic administration [104], but more research is needed to operationalize and investigate how best to incorporate these factors into treatment protocols. Set is typically defined as the mindset, psychosocial education, and experience that a participant brings with them as they enter treatment, and setting is defined as the environment in which the psychedelic compound is administered. The dependence of the clinical and psychological effects of psychedelics on the mindset and environment of the user suggests that they truly are “experiential medicines”. Increasingly, human studies with psychedelics are attempting to systematically modify set and setting, either to study set and setting as independent variables affecting the outcomes of the study (NCT04410913), or by making them a fixed key part of the study design, such as electing to use group psychotherapy and/or drug administration instead of individual sessions [105, 106]. Group treatment processes likely result in qualitatively different therapeutic environments that differ from individual treatments in ways beyond economics and scalability. The long history of the group use of psychedelic substances in Indigenous and other traditional settings across North and South America suggests that, with the proper context and training, it is possible for group psychedelic experiences to be safely managed and to result in positive outcomes for the participants [107, 108]. Nevertheless, most of the clinical data to date have been generated using a fairly homogenous clinical approach, and so specific experiments should be conducted with more variation in set and setting to determine how best to potentiate the therapeutic value of these variables, while mitigating possible harm when administering psychedelics as medical therapies.

## Optimizing set while maintaining blinding

One concern that is repeatedly raised in the discussion of psychedelic trials is the conundrum around experimental blinding [109]. The prevailing belief is that psychedelic trials are difficult to blind and therefore one must always worry that expectation is coloring outcome. This is especially true when participants enroll in a trial that not only takes up weeks of their life, but also assesses whether the investigational compound occasions a particularly meaningful, and often spiritual, life experience. While maintaining a double-blind is indeed a common challenge in psychedelic trials, several methods can be utilized to minimize the impact of expectation and ensure that clinical outcome measures reflect the long-term and durable effects of psychedelic therapies. For example, the use of an active control drug and/or a psychedelic naïve subject population may make it more difficult for even a well-informed participant to be confident of their treatment assignment. In addition, the use of a centralized assessment core to evaluate outcome measures ensures that data collection is blinded and homogeneously collected across study sites while also mitigating the risk of participants inflating their improvements to please study staff with whom they have developed a therapeutic alliance. Perhaps most importantly, the collection of long-term follow-up data from study participants can partially address concerns regarding expectation effects and can speak to the potential durability of psychedelic-induced change. While it is reasonable to suggest that a participant in the throes of a clinical trial might inadvertently exaggerate their improvements when surrounded with engaged and supportive staff, it is less reasonable to assume that this effect would last months after the trial has ended and the participant is again immersed in their regular environment.

## Limitations and future directions for psychedelic therapies

The recent explosion in interest in psychedelic therapies has been based on multiple preliminary reports suggesting the potential of safety and efficacy in various psychiatric and general medical conditions, especially mood disorders, alcohol use disorder, and PTSD. These data, however, are not without their limitations. As clinically effective as psychedelics can be when administered under the right conditions, it would be negligent to forego mention of the study participants who do not respond discernably to psychedelic agents. For example, while the recent phase 3 trial of MDMA therapy for PTSD showed that 67% of participants gain complete remission from PTSD, and another 21% exhibited a clinically meaningful response, this still left 12% of study participants with no clinically meaningful response. Similarly, a recent phase 2 trial of psilocybin therapy for an episode of treatment-resistant depression showed that, while 37% of participants displayed a clinically meaningful response to psilocybin at the primary endpoint (week 3), most did not [56]. While some of this can perhaps be chalked up to the impact of set and setting, some of it is undoubtedly due to differences in sensitivity to psychedelic compounds, and perhaps also to differences in the response to the uncertainty and change brought about by these therapies. In addition, genetics play a role not only in pharmacokinetics but also in suggestibility and the development and maintenance of emotional memories [110] and may therefore also impact the effects of psychedelic therapies. One would hope that, as precision medicine advances, and as adaptive trials and genetic testing enable us to better tailor treatments to individual patients, biological and behavioral factors will be used to ensure that the potential therapeutic impact of psychedelic therapies is maximized.

Psychedelics are powerful compounds that are capable of enabling great change. As such, they should be approached with care and caution. Under the best of circumstances, and when properly facilitated, psychedelic therapy can kindle the release of some of the most deeply entrenched negative affective states and thought processes, resulting in clinical recovery and positive growth. However, the experiential flipside is equally relevant: occasionally, and especially when taken under suboptimal conditions, without adequate support, or at too high a dose, psychedelics can trigger dysphoria, disorganize thought, and spark delusional perceptions [111–114]. In addition, given the largely explanatory trials dataset available to date, it remains to be seen how clinical outcomes will be shaped by different real-world factors such as personality disorders, significant psychiatric and medical comorbidities, and the combination of psychedelics with different behavioral therapies or even with other psychedelics (e.g., psilocybin plus MDMA). We must therefore move forward with care and forethought. These compounds may potently manifest aspects of the human psyche in a manner that can both help and harm. As such, scientific investigations into the judicious use of psychedelics test our capacity for, and professional commitment to, the proper uses of clinical power in the service of healing. It is time that psychedelic therapies be carefully reconsidered [115].
